# Telepsychiatry for the Elderly

**DOI:** 10.1192/j.eurpsy.2022.186

**Published:** 2022-09-01

**Authors:** G. Stoppe

**Affiliations:** University of Basel, Mentage, Basel, Switzerland

**Keywords:** old age psychiatry work force, helper conference, cultural sensitivity, access

## Abstract

For many old people with mental health problems, access to psychotherapeutic and psychiatric help is often difficult. This is partly because going to a psychiatrist is still stigmatised, especially among the older generation. On the other hand, therapists with an interest in and competence for older people are often not sufficiently available even in the well-supplied western countries. In this situation, digitalisation offers various opportunities. Basically, the internet is a good way to promote health literacy. Classic psychoeducation can certainly be offered on the internet. And psychotherapy can also be administered with the help of the internet. Especially in the COVID-19 pandemic, the possibilities of internet-based therapies, for example Zoom or other techniques, were practised. This means that people with limited mobility can also receive therapy over long distances. This technology also makes it possible, for example, for the migrant population to receive therapy in their national language. All these possibilities are under development, but may become routine in the future. With the help of the digital possibilities, it is possible to organise helper conference. The professional exchange between relatives, family doctors, psychiatrists and other people in the help system can be easily organised in this way. The method also saves travel time, which is often not reimbursed in the health systems.

**Disclosure:**

No significant relationships.

